# Rapid ice-marginal lake growth in Alaska driven by glacier retreat through bed overdeepenings

**DOI:** 10.1073/pnas.2513289123

**Published:** 2026-03-09

**Authors:** Daniel McGrath, Louis Sass, William H. Armstrong, Caitlyn Florentine, Scott W. McCoy

**Affiliations:** ^a^Department of Geosciences, Colorado State University, Fort Collins, CO 80523; ^b^U.S. Geological Survey Alaska Science Center, Anchorage, AK 99508; ^c^Department of Geological and Environmental Sciences, Appalachian State University, Boone, NC 28608; ^d^U.S. Geological Survey Northern Rocky Mountain Science Center, Bozeman, MT 59717; ^e^Department of Geological Sciences and Engineering, University of Nevada, Reno, NV 89557

**Keywords:** glaciology, glacial lakes, climate change

## Abstract

The number and cumulative area of ice-marginal lakes have expanded globally in recent decades, with many lakes residing in glacier-bed overdeepenings, which are subglacial basins formed through preferential glacial erosion. However, current lake expansion rates, key drivers of expansion, and maximum future lake extents are poorly quantified. This is notable because glacial lakes pose hazards, alter hydrologic and ecological systems, and, in some cases, accelerate glacier flow and retreat. Here, we quantify recent ice-marginal lake growth and use existing ice thickness and topographic data to map glacier-bed overdeepenings in Alaska as a predictor of recent and future locations of lake growth. Ice-marginal lakes in the region grew by +156 km^2^ (26 km^2^ y^−1^) between 2018 and 2024, representing a 50% increase relative to the 2009–2018 rate. Eighty percent of lake growth since 2018 occurred in mapped glacier-bed overdeepenings. Approximately 4,250 km^2^ (2,966 to 5,503 km^2^ accounting for ± ice thickness uncertainty) of the overdeepened area is connected to an ice-marginal lake, indicating the potential for more than fourfold lake growth of existing lakes as glaciers retreat. An additional 14,500 km^2^ (12,469 to 17,134 km^2^) of remaining glacier area resides on glacier-bed overdeepenings not connected to existing lakes, highlighting the potential for substantial new lake development. Velocities from lake-terminating glaciers show clear passive and dynamic endmembers on a continuum of glacier–lake coupling. Glaciers with ice-marginal lakes thinned 23 to 54% more than glaciers of similar area without lakes, underscoring the critical importance of dynamic glacier–lake coupling on the evolution of glaciers in Alaska.

## Significance

Glacial lakes in the Alaska region expanded by >150 km^2^ between 2018 and 2024, representing a 50 to 120% faster rate of expansion than previous periods (1986–2018). This growth primarily occurred in mapped glacier-bed overdeepenings. There is approximately 4,250 km^2^ of glacier-bed overdeepenings connected to lakes today, indicating that future regional ice-marginal lake area could be more than four times larger than today. Glacier response to ice-marginal lakes varies substantially as some exhibit a dynamic response with positive feedbacks leading to glacier retreat and rapid lake growth, while others show a passive response, where lake growth is typically much slower. Understanding this response is critical for projecting individual glacier mass loss and lake growth.

Glacier-bed overdeepenings are ubiquitous beneath ice masses where concentrated glacial erosion deeply incises sediment or bedrock to generate topographic depressions (1–6). Overdeepenings strongly influence subglacial hydrology, ice dynamics (7–9), and vulnerability to ocean-driven retreat (10). As glaciers retreat, water can fill these topographic depressions to form glacial lakes (e.g., refs. 11 and 12), as long as the volumetric sediment supply is less than the rate of ice loss within the overdeepening.

Globally, the number, area, and volume of glacial lakes have expanded since the 1990s (13, 14). The greatest number of glacial lakes are found around the periphery of the Greenland Ice Sheet, in High Mountain Asia, Western Canada and United States, and Alaska (14).

As of the 2016–2019 interval, there were more than 650 lakes larger than 0.05 km^2^ within 1 km of glaciers in Alaska, cumulatively covering nearly 1,300 km^2^ (15). Lake number and area were dominated by moraine-dammed lakes, followed by bedrock lakes and supraglacial lakes (15). Between ~1986 and ~2018, lake area increased by 483 km^2^, with the greatest growth in moraine-dammed proglacial lakes. A focused study examining a subset of 107 lakes in Alaska found that lake area increased by 58% between 1984 and 2018 and that lake growth most commonly occurred proximal to large glaciers with wide, thick ice adjacent to the lake (16). Ice-marginal lakes can impact the rate of mass loss of upstream glaciers, with Alaska (17) and Patagonia (18) lake-terminating glaciers losing >6 Gt y^−1^ to iceberg calving and subaqueous melt. This is one contributing mechanism to the high rate of mass loss in the Alaska region (–60.8 Gt y^−1^, 2000–2023) which represents the highest rate of any glacierized region external to the ice sheets globally and constitutes ~22% of total global glacier mass loss (19).

Given the significant potential impact of proglacial lakes on glacier dynamics and hazards, numerous studies have sought to predict where new glacial lakes might form in the future (e.g., refs. 11 and 20). These studies have identified glacier-bed overdeepenings as likely locations for future lake formation, so long as retreat through the overdeepening outpaces sediment infill. However, few studies have quantified the historical relationship between glacier-bed overdeepenings and glacial lake growth, which is a critical foundation for mapping potential future lake growth and identifying specific glaciers/regions vulnerable to rapid lake expansion, and hence inform hazard and glacier mass loss assessments.

In this study, we i) quantify changes to ice-marginal lakes in Alaska between 2018 and 2024 using Sentinel-2 optical imagery, and ii) map glacier-bed overdeepenings by differencing modeled ice-thickness estimates (21) from surface elevations. We find a strong correlation between areas of lake growth and mapped glacier-bed overdeepenings. Such a correlation implies that mapped bed overdeepenings can be used to determine where lake growth will cease, where lakes may grow substantially in the future, and identify glaciers harboring future lake basins. Critically, the rate of lake growth depends on the degree to which the lake alters ice dynamics. Using glacier velocity data (22), we provide examples of two modes of glacier–lake coupling: a dynamic mode where the lake triggers a perturbation to the glacier force balance, leading to ice flow acceleration, increased calving, and glacier retreat, leading to further lake expansion, and a passive mode, where the presence of the lake does not alter ice dynamics at present (i.e., stable or slowing near-terminus ice velocities) and lakes grow passively. A systematic study of individual lake-glacier systems is required to accurately assess the dynamic sensitivity of glaciers to lake expansion and predict future lake growth rates.

## Ice-Marginal Lake Growth

**Fig. 1.**
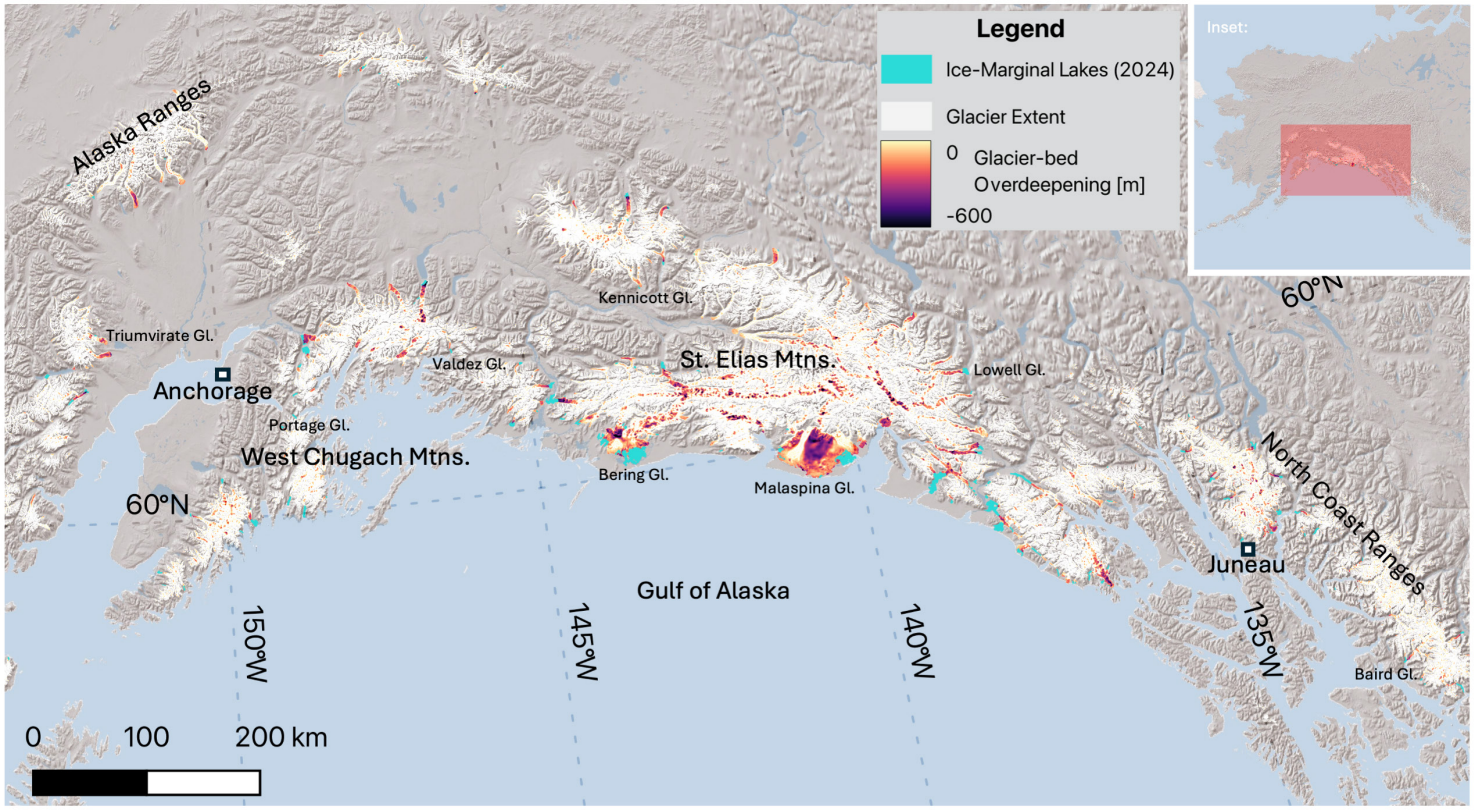
Overview map showing ice-marginal lakes, glacier extent, and mapped glacier-bed overdeepenings. The *Inset* shows study location in North America. Named glaciers are shown in Fig. 2. Hillshade Copyright:© 2014 Esri. Map projection is NAD83/Alaska Albers (EPSG: 3338).

Ice-marginal glacial lakes (>0.5 km^2^) in Alaska (Fig. 1) had a net area change of +156.4 ± 11.5 km^2^ between 2018 and 2024, the result of 203.7 km^2^ (n = 118) of net area growth and –47.3 km^2^ (n = 29) of net area loss. Moraine-dammed proglacial lakes experienced the greatest cumulative net growth, while debris-dammed and ice-dammed lakes were more variable, with a cumulative net loss in area for ice-dammed lakes. This yields an annual area increase rate of 26.1 km^2^ y^−1^, a 120% increase relative to the 1986–1999 rate of 11.7 km^2^ y^−1^, and an approximately 50% increase relative to the 1999–2009 and 2009–2018 rates of 17.7 and 17.1 km^2^ y^−1^, respectively (*SI Appendix*, Fig. S1*A*).

**Fig. 2.**
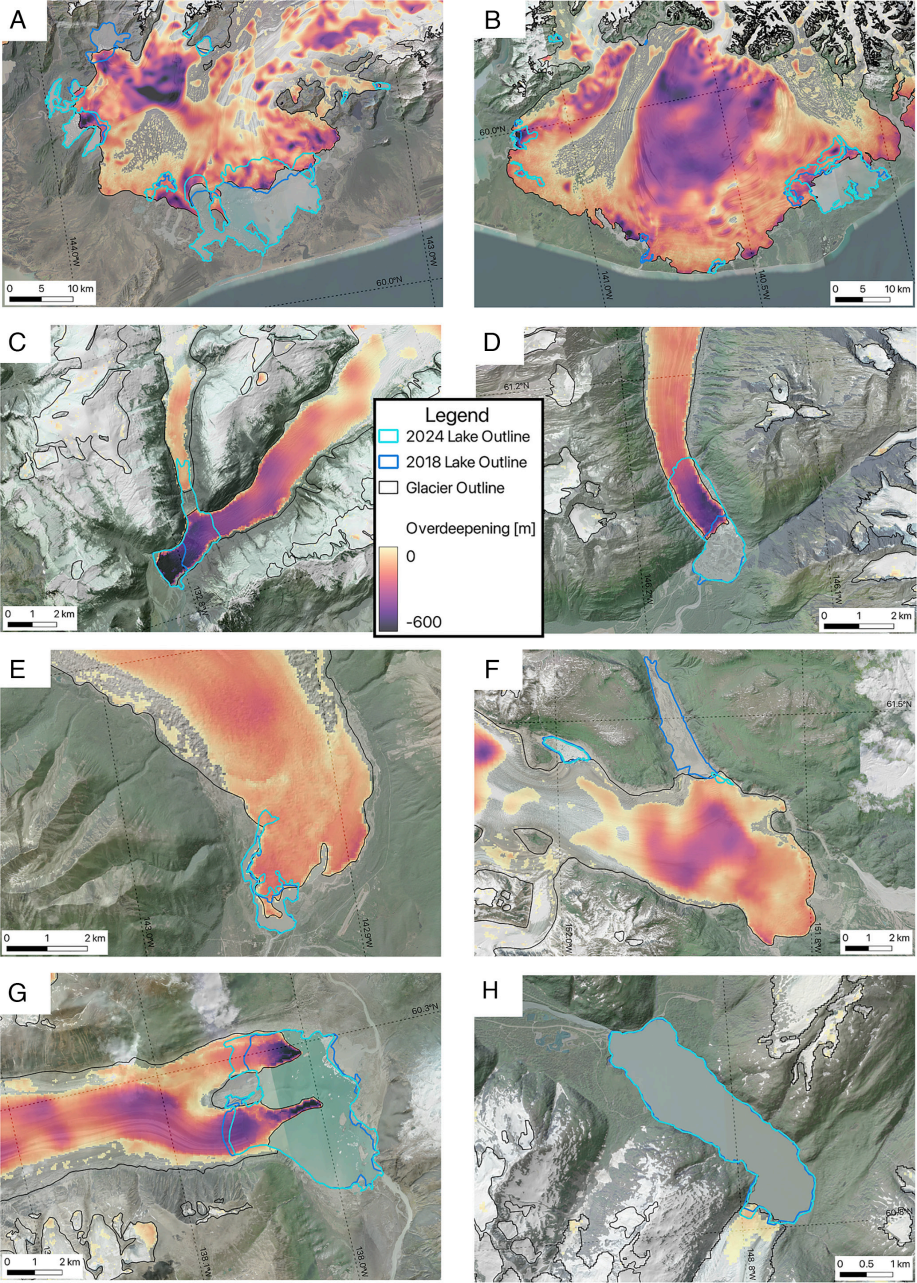
Mapped glacier-bed overdeepenings and ice-marginal lake change between 2018 and 2024 overlaid on hillshade enhanced optical imagery for eight glacier–lake systems that typify patterns in observed lake area change. Glaciers include (*A*) Bering, (*B*) Malaspina, (*C*) Baird, (*D*) Valdez, (*E*) Kennicott, (*F*) Triumvirate, (*G*) Lowell, and (*H*) Portage Glaciers. Note spatial scales vary between images. Imagery source: Esri, Vantor, Earthstar Geographics, and the Geographic Information System (GIS) User Community. Hillshade source: U.S. Geological Survey (USGS) National Map 3D Elevation Program (3DEP).

More than half of the net ice-marginal lake growth occurred in just two lakes (Vitus Lake and Malaspina Lake) associated with two piedmont glacier complexes: Bering and Malaspina (*Sít’ Tlein;* Seward and Agassiz) Glaciers (Fig. 2 *A* and *B* and *SI Appendix*, Fig. S1 *B* and *C*). The mechanisms for lake growth varied across the region: In some instances, entire glacier tongues disintegrated, and the termini retreated 1.5 to 2 km during this period (e.g., Ellsworth, Baird, and Valdez Glaciers, Fig. 2 *C* and *D*). Elsewhere, supraglacial lakes expanded and coalesced, sometimes merging with proglacial lakes (e.g., Kennicott Glacier, Fig. 2*E*).

Lake-area loss was dominated by ice-dammed lakes that either completely or partially drained between 2018 and 2024, including lakes proximal to Steller (–22.2 km^2^), Triumvirate (–4.5 km^2^; Fig. 1*F*), and Fairweather (–2.7 km^2^) Glaciers. Ice-dammed lakes are typically more dynamic than moraine-dammed lakes and can fill/drain on subyearly timescales (23). In other locations, debris-dammed lakes drained, some of which had likely formed due to past glacier surges (e.g., Donjek Glacier, –1.9 km^2^). Changing delta dynamics, stage lowering, or modest terminus advances (e.g., Lowell Glacier; Fig. 2*G* and *SI Appendix*, Fig. S2), also contributed to lake area loss. Approximately 20% of lakes remained stable, defined as less than ±0.1 km^2^ of change, including the lake proximal to Portage Glacier (Fig. 2*H*).

## Glacier-Bed Overdeepenings Accommodate Lake Growth

Glacier-bed overdeepenings are large-scale subglacial topographic depressions formed through preferential bed erosion likely tied to positive feedbacks related to subglacial hydrology (1–6). Where these overdeepenings are proximal to the modern glacier terminus, glacier retreat can lead to either lake formation or lake growth as long as sedimentation rates are low enough to avoid infilling as the glacier retreats. Where these overdeepenings are found further up-glacier, not directly adjacent to the glacier terminus, they represent potential lake basins following future glacier retreat (e.g., ref. 11).

In total, 1,171 glaciers in the Alaska region have glacier-bed overdeepenings that exceed 0.5 km^2^ in area, with a cumulative surface area of 14,497 km^2^ (12,469 to 17,134 km^2^ accounting for ± ice thickness uncertainty) and volume of 1,667 km^3^ (882 to 2,696 km^3^ accounting for ± ice thickness uncertainty). More than 40% of cumulative overdeepened ice volume and more than 25% of cumulative overdeepened ice area is found in just five glaciers: Seward [357 km^3^ (21%); 1,722 km^2^ (12%)], Bering [145 km^3^ (9%); 1,067 km^2^ (7%)], Hubbard [85 km^3^; (5%), 557 km^2^ (4%)], Steller [57 km^3^ (3%); 264 km^2^ (2%)], and Agassiz [49 km^3^ (3%); 377 km^2^ (3%)] Glaciers, all of which have large ice-marginal lakes. These glaciers are also some of the largest in the region, accounting for 12.5% of total glacierized area. Of the ~1,100 glaciers with overdeepenings >0.5 km^2^, the median percent of overdeepening area is 13% (mean = 16%), but a few notable glaciers, including east Yakutat and Seward Glaciers are overdeepened for 55% and 51% of their total area, respectively, indicating that these glaciers will likely be interacting with lakes for much of their future evolution.

**Fig. 3.**
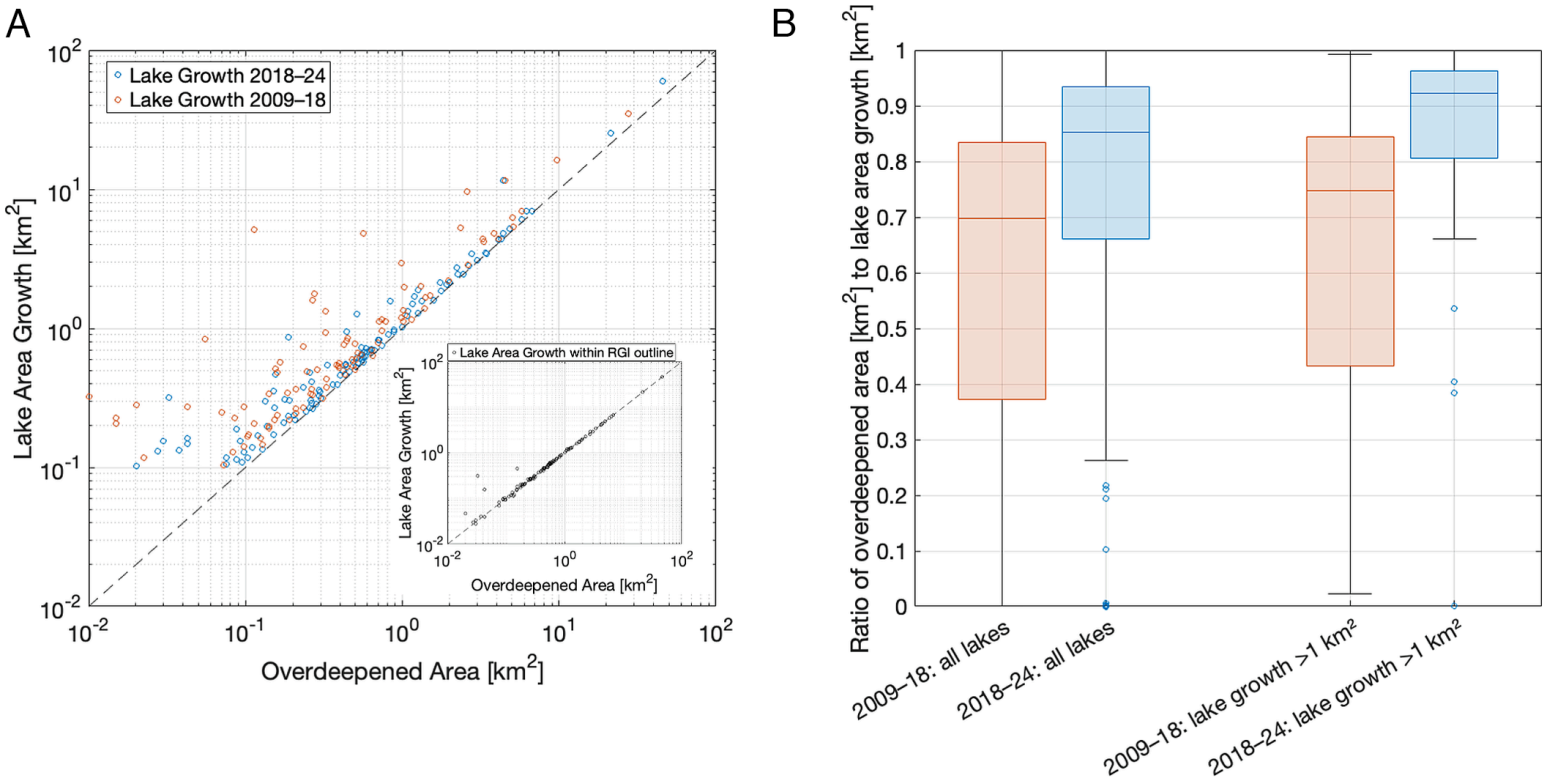
Ice-marginal lake growth has predominantly occurred within mapped glacier-bed overdeepenings. (*A*) Log–log plot of lake area growth (km^2^) vs. overdeepened area (km^2^) for all lakes that experienced >0.1 km^2^ of growth during each interval. *Inset*: Log–log plot of lake area growth that occurred within RGI glacier extents vs. overdeepened area between 2018 and 2024. For example, this excludes lake growth where an ice-dammed lake forms in a nonglaciated tributary valley or where the 2018 glacier terminus position was outboard of the mapped RGI extent used to generate ice thickness estimates (21). (*B*) Boxplots of the ratio of mapped overdeepened ice area to lake area growth for each interval for all lakes and lakes that experienced >1 km^2^ of growth. Box and whisker plots show standard data statistics: boxes extend to first quartile and third quartile, the horizontal line is the median, and whiskers extend to 1.5× the interquartile range. A value of 0.8 indicates that 80% of the lake growth occurred within a mapped glacier-bed overdeepening.

Mapped glacier-bed overdeepenings are a primary control on ice-marginal lake growth over the past 15 y (r^2^ = 0.92 (*P* < 0.05) and 0.99 (*P* < 0.05) for 2009–2018 and 2018–2024, respectively; Fig. 3*A*). In total, 63% and 80% of lake area growth, between 2009–2018 and 2018–2024, respectively, occurred in mapped glacier-bed overdeepenings, although this is conservative for certain lakes where mapped ice thicknesses did not match 2018 lake margins (e.g., Bering Glacier, Fig. 2*A*) or lake growth occurred in an ice-dammed tributary valley (e.g., Tazlina Glacier). If only lake area growth within the mapped glacier outlines (24) is considered for the 2018–2024 period, this increases to 99%, indicating that nearly all lake area growth due to changes in glacier extent occurred within mapped glacier-bed overdeepenings (Fig. 3 *A*, *Inset*). At the individual lake scale and where lake growth exceeded 1 km^2^, the median ratio of overdeepened ice area to lake area growth was 0.75 (2009–2018) and 0.92 (2018–2024) (Fig. 3*B*). For the most recent interval, lakes that grew by more than 1 km^2^ accounted for only 25% of the total lake number but 84% of total lake area growth. Collectively, those observations show that the dominant pattern of lake growth in Alaska is into mapped glacier-bed overdeepenings and that the modeled ice thicknesses (21) accurately capture the spatial extent of these overdeepenings. Furthermore, these findings illustrate that the rate of glacier retreat combined with the magnitude of the overdeepenings far outpaces the sediment flux into these basins, as we found limited evidence for mapped overdeepenings transitioning to sediment-filled basins.

## Future Lake Growth

The strong relationship between glacier-bed overdeepenings calculated from the Millan et al. (21) ice thickness estimates and ice-marginal lake growth in the recent past illustrates that, despite known errors and limitations in ice thickness estimates (25, 26), the mapped glacier-bed overdeepenings provide foundational insight into the topographic controls on future lake growth. In total, the area of mapped glacier-bed overdeepenings connected to a modern lake is 4,268 km^2^ (2,966 to 5,503 km^2^ accounting ± ice thickness uncertainty; *SI Appendix*, Fig. S3). Total ice-marginal lake area in this study (2024) is 1,247 km^2^, so the bed topography that will be exposed as glaciers retreat offers the capacity for existing lakes to expand in area by more than fourfold. Lakes with the greatest potential expansion are the Malaspina Glacier complex (Agassiz/Seward Glaciers) which contains >1,500 km^2^ of connected glacier-bed overdeepened area, as well as the Bering/Steller Glacier complex (566 km^2^). Airborne radar campaigns on Malaspina Glacier (25) have shown that modeled ice thickness estimates (21) significantly overpredict ice thickness. Nonetheless, the area of mapped overdeepenings for Malaspina Glacier computed with ice thickness from an airborne radar campaign (25) (1,390 km^2^) falls within the range of glacier-bed overdeepening area (1,307 to 1,748 km^2^) computed in this study accounting for ice thickness uncertainty (21). Other glacial lakes with large growth potential (150 to 200 km^2^) into glacier-bed overdeepenings include Brady, east Yakutat, and Vern Ritchie/Battle Glaciers. Large overdeepenings such as these underlie the projected persistence of ice–lake contact for the next 74 y (38 to 177 y) on the region’s lake-terminating glaciers (17).

This analysis also identifies lakes that have experienced recent growth through mapped glacier-bed overdeepenings but whose current lake margins are approaching the maximum extent of this area (e.g., Ellsworth Glacier; *SI Appendix*, Fig. S4*A*). This implies that further glacier retreat will transition the glacier from a lake to land-terminating glacier, ending lake growth. These examples are limited, however, and 119 lakes (81%) have >1 km^2^ of growth potential.

Despite the strong correlation between lake growth and glacier-bed overdeepenings, we identify notable exceptions where the presence of an overdeepened bed proximal to an ice-marginal lake has not resulted in significant retreat. A prominent example is the proglacial lake at Tulsequah Glacier (*SI Appendix*, Fig. S4*B*), where lake area remained stable between 2018 and 2024 yet the lake is adjacent to >16 km^2^ of overdeepened bed. This highlights the need for additional research to identify what processes (e.g., surface mass balance, frontal ablation dynamics, valley constrictions, sedimentation rates, and/or bed geometry) have contributed to the quasi-stability of this system.

## Dynamic vs. Passive Lake Growth

Our mapped glacier-bed overdeepenings identify areas prone to future lake growth, but the rate of future lake growth requires detailed consideration of glacier ice dynamics. Ice-marginal lakes can influence glaciers through a combination of thermal and mechanical processes (27, 28) leading to frontal ablation and enhanced flow speeds relative to land-terminating glaciers (29, 30). However, near-terminus ice velocities for lake-terminating glaciers show much larger variability than land-terminating glaciers (30), illustrating the complexity of glacier–lake interactions and dependence on a host of additional factors (7, 9, 16, 28, 29, 31, 32) (e.g., ice thickness, lake depth, water temperature, valley width, surface and bed slope, lateral vs. basal drag distribution, subglacial hydrology).

There is a spectrum of ice-marginal lake coupling with ice dynamics; we illustrate the fully dynamic and fully passive endmembers of the spectrum using satellite-derived ice surface velocity time series (22). Detailed observations (e.g., annual terminus positions) and additional research would be required to ascertain where and why each of the region’s lake-terminating glaciers falls on this spectrum.

The dynamic response mode consists of an initialization phase and an arrest phase. In the initialization phase, ice velocities increase through dynamic feedbacks. This occurs when the geometry of the glacier-bed overdeepening is such that glacier thinning decreases the resistive stresses more than it decreases the driving stress, and the glacier accelerates. This mechanism is similar to a tidewater glacier, but typically more subdued, possibly due to the thermal limitations on subaqueous ablation in a cooler-water lake setting (33, 34). However, lake-terminating glaciers can still have high frontal ablation rates (17, 18), and can develop floating tongues, or active calving fronts (e.g., refs. 35 and 36).

**Fig. 4.**
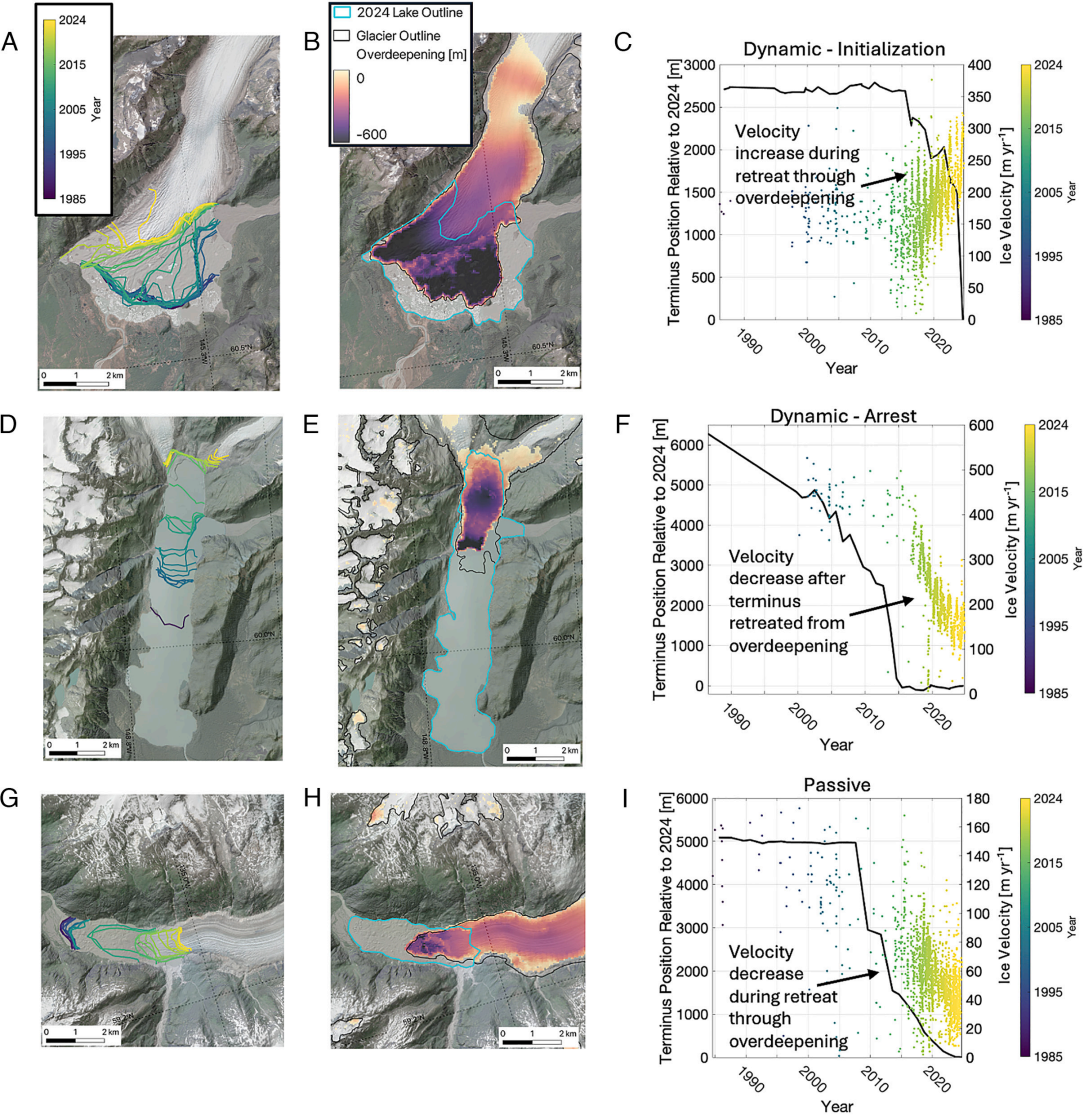
Glacier–lake systems are coupled through two modes: dynamic and passive. (*A*) named anchor Terminus positions named anchorfrom 1985–2024, (*B*) 2024 lake outlines and mapped named anchorbed-overdeepenings, and (*C*) Terminus position relative to 2024 (m) and Ice velocity (m y^−1^) for Sheridan Glacier, (*D*) Terminus positions from 1985–2024, (*E*) 2024 lake outlines and mapped bed-overdeepenings, and (*F*) Terminus position relative to 2024 (m) and Ice velocity (m y^−1^) for Excelsior Glacier, (*G*) Terminus positions from 1985–2024, (*H*) 2024 lake outlines and mapped bed-overdeepenings, and (*I*) Terminus position relative to 2024 (m) and Ice velocity (m y^−1^) for Meade Glacier. Sheridan (*A*–*C*), Excelsior (*D*–*F*), and Meade (*G–I*) Glaciers illustrate dynamic mode initialization phase, dynamic mode arrest phase, and passive mode interactions between ice velocity and lake growth, respectively. Point colors in the velocity time series (*Right*-most panels) correspond with the associated terminus position in the map-view time series (*Left*-most panels). Imagery source: Esri, Vantor, Earthstar Geographics, and the GIS User Community. Hillshade source: USGS National Map 3DEP.

In the initialization phase, the increase in ice velocity is synchronous or nearly synchronous with increased terminus retreat. For example, the western margin of Sheridan Glacier remained relatively stable through 2015 (Fig. 4*A*). As the terminus pulled away from the valley walls, median annual ice velocities increased by 40% from 160 to 230 m y^−1^ between 2015 and 2024 (Fig. 4*C*). Sheridan Glacier has >9 km^2^ of connected ice within overdeepenings, indicating that continued lake growth is likely. Other glaciers exhibiting this behavior between 2018 and 2024 include Alsek and Baird Glaciers (*SI Appendix*, Fig. S5 *A* and *B*), which retreated by 2.6 and 1.6 km, respectively, while median annual ice velocities increased by 86% and 71%, respectively, over this interval. Additionally, Bear and Bainbridge Glaciers had notable dynamic responses (*SI Appendix*, Fig. S6) and have evidence of ocean-water intrusions into the proglacial lakes at their terminus resulting in brackish or even salty lagoons (e.g., ref. 37). Ocean-water intrusions increase heat transport and water density in the lake, which can affect subaqueous melt rates and buoyant forces at the glacier terminus. Therefore, these systems are a hybrid between a typical tidewater glacier system and lake terminating systems.

In the arrest phase of the dynamic mode, the mechanism for decreasing resistive stresses is removed as the ice retreats out of the glacier-bed overdeepening, while thinning continues to decrease the driving stress. The decrease in ice velocities is synchronous or nearly synchronous with stabilization of the terminus position (i.e., a decrease in retreat rate). This is typically coincident with retreat out of the glacier-bed overdeepening but also appears to occur mid-overdeepening in some cases, likely due to valley constrictions or bed topography. Excelsior Glacier is a clear example of this process (Fig. 4 *D–F*), with velocities prior to 2016 of ~400 m y^−1^ associated with rapid lake growth, and a decrease to ~150 m ^−1^ in 2024 after lake growth ceased. Ellsworth Glacier and an unnamed glacier (RGI 6.0 – 01.05638) further illustrate the dynamic arrest mode, where ice velocities are declining following a period of lake growth and rapid ice flow (*SI Appendix*, Fig. S5 *C* and *D*). For the latter example, velocities declined by >60% between 2018 and 2024, following termination of lake growth.

In the passive glacier response mode, ice velocities either remain stable or decrease synchronously with terminus retreat. The glacier-bed overdeepening accommodates lake growth but does not seem to alter or influence flow processes to enhance glacier retreat. This is most often associated with shallow lakes, low velocity and low slope glaciers (e.g., piedmont lobes), or glaciers where the terminus is positioned in a lateral topographic constriction. Commonly, glaciers in these instances have low driving stresses and slow velocities, and downstream ice provides limited resistive stresses. Lake growth may still occur, but subaqueous ablation and frontal ablation are limited relative to the surface mass balance, and lake growth does not significantly modify the force balance. As an example, Meade Glacier retreated by ~3 km between 2009 and 2024 (Fig. 4 *G–I*) while the near-terminus annual median ice velocity synchronously decelerated from 96 to 38 m y^−1^. Other examples of passive lake growth are Malaspina and Kennicott Glaciers, where ice velocities have remained stable or modestly declined (Fig. 2 and *SI Appendix*, Fig. S5*E*). Valdez Glacier is a particularly illustrative example given that its terminus retreated substantially (~2 km) between 2018 and 2024 (Fig. 2*D*) yet there was no glacier velocity response over that time (*SI Appendix*, Fig. S5*F*). It is possible that the terminus was thin, near flotation, and thus provided limited resistive stress prior to calving. This demonstrates how retreat, and associated lake growth, may not alter the near-terminus force balance, resulting in a passive glacier response.

Comparing the range of dynamic coupling observed between lakes and lake-terminating glaciers in Alaska with other regions of the globe illustrates the influence of heat transfer and frontal ablation on glacier dynamics. Patagonia has glaciers flowing into large (>1,000 km^2^), deep (>500 m), warm lakes (31), some of which have near-terminus velocities greater than 1,000 m y^−1^ (38). In comparison, the lakes in Alaska are much colder (28) and even frozen for large parts of the year, which, combined with smaller (15) and shallower lakes, results in much lower potential heat transfer and frontal ablation rates. In the Himalaya Mountains, lake temperatures are generally intermediate to those in Alaska and Patagonia (39), but the lake size tends to be smaller and shallower than most of the lakes that have been studied in Patagonia and Alaska. The near-terminus velocities tend to be low (<10 m y^−1^) (40), likely because lower rates of heat transfer limit frontal ablation. Nevertheless, glaciers in the eastern Himalaya Mountains, with larger and more developed lakes, show a dynamic response with multidecadal increases in ice velocity (40). These comparisons illustrate the broad spectrum of glacier–lake coupling, highlighting the need for detailed study at both the individual lake/glacier scale, as well as across regions.

## Implications and Conclusions

Glaciers in Alaska are losing the greatest cumulative mass of any glacierized region on Earth external to the ice sheets, characterized by a specific mass-change rate (–0.72 m w.e. y^−1^) that is nearly double the global average (–0.41 m w.e. y^−1^) from 2000 to 2023 (19). Alaska also saw the greatest growth in glacial lake area of any subregion between 1990 and 2020 (14). Previous work in Alaska (41) and in other glacierized regions [e.g., Himalaya Mountains (29)] have shown that lake-terminating glaciers have more negative specific mass balances than land-terminating glaciers. Analyzing published ice thinning rates (42), we find that for glaciers in Alaska that have ice-marginal lakes >0.5 km^2^, the median 2010–2019 thinning rate of –1.15 m y^−1^ was 0.64 m y^−1^ more negative than glaciers that did not have ice-marginal lakes (–0.51 m y^−1^; *SI Appendix*, Fig. S7*A*). However, part of this difference is simply caused by the difference in median glacier size (0.3 km^2^ for all glaciers vs. 57.8 km^2^ for lake-terminating glaciers in this study; *SI Appendix*, Fig. S7*B*) because larger glaciers are thinning faster than smaller glaciers in the Alaska region. Accounting for glacier area using size classes (1 to 10, 10 to 50, 50 to 100, >100 km^2^), we still find that glaciers with ice-marginal lakes >0.5 km^2^ have median thinning rates that consistently exceed similar sized glaciers without ice-marginal lakes. These differences were statistically significant for 10 to 50, 50 to 100, and >100 km^2^ size classes, where glaciers with ice-marginal lakes thinned more than glaciers without ice-marginal lakes by –0.36 (34% difference), –0.26 (23% difference), and –0.53 m y^−1^ (54% difference), respectively (*SI Appendix*, Fig. S7). These elevated thinning rates suggest that overall, mass loss through frontal ablation and/or dynamic coupling to up-glacier ice is prevalent enough to alter the average mass balance signal, as these region-wide comparisons would presumably be statistically comparable if coupling was dominated by the passive mode of lake growth (i.e., dominantly forced by surface mass balance with minimal dynamic feedbacks).

Our findings show that ice-marginal lake growth was closely correlated with glacier retreat through glacier-bed overdeepenings, providing a simple explanation for historical growth and a tool for identifying lakes/glaciers that may experience rapid change in the coming years to decades. We find that connected glacier-bed overdeepenings are more than four times larger than present lake area, indicating that a fourfold increase in regional lake area is possible as these glaciers retreat. For the two largest piedmont glacier systems in the region, future lakes could encompass between 1,307 to 1,748 km^2^ (Malaspina) and 360 to 950 km^2^ (Bering). The former would be the second largest lake in Alaska behind Iliamna Lake. In total, there are more than 20 lakes currently connected to glacier-bed overdeepenings (i.e., potential lake basins) that exceed 25 km^2^ in area.

We describe two endmembers of lake growth: dynamic and passive. In the dynamic mode, the formation and growth of proglacial lakes perturb the glacier force balance leading to near-terminus acceleration and enhanced thinning and retreat in a positive feedback loop. Identifying glacial-lake systems that are prone to this dynamic response requires detailed study of each system, but the mapped glacier-bed overdeepenings presented in this study can guide these critical investigations. Lake growth has far-reaching implications, influencing downstream hydrologic dynamics (43), habitat quality (44), glacial lake outburst hazards (45), and even atmospheric CO_2_ concentrations (46). Given rates of lake growth in recent years, coupled with the potential for future growth across this area, continued study of these systems is required to assess ecological impacts and hazard assessments.

## Materials and Methods

Using an existing glacial lake inventory (15, 47), we identified glacial lakes in Alaska greater than 0.5 km^2^ (n = 211) for the period 2016–2019 (median year 2018). Using Sentinel-2 optical imagery from 1 May to 30 September 2018 and 1 May to 30 September 2024, we manually mapped lake extents at 1:10,000 scale for these 2 y (48) in the open-source QGIS software program version 3.36.3-Maidenhead (49). We estimated error in lake area by multiplying the lake perimeter (in km) by half the Sentinel-2 pixel resolution (5 m or 0.005 km). We also reviewed and updated the lake connection category (15), which describes the spatial relationship between a lake and its source glacier. These categories include: proglacial (lakes at terminus of glacier, in contact with the ice), supraglacial (lakes on surface of glacier ice), ice (ice-dammed lakes found at ice margins or tributary valleys), detached (lakes fed by glaciers but not in contact with the ice), or unconnected (detached lakes that are not fed by glaciers). This study solely assesses ice-marginal lakes (n = 147 lakes), defined as having contact with a glacier and thus excludes detached and unconnected lakes.

We computed two area metrics for each lake: lake-area growth and net change (*SI Appendix*, Fig. S2). Lake-area growth (km^2^) is the area sum of all pixels where the lake expanded but does not account for lake area lost. Net change (km^2^) is the sum of lake-area growth and lake-area loss per lake. Lake-growth metrics are plotted in Fig. 3. In a few instances, lakes expanded and merged with other existing lakes from the 2018 inventory. For these lakes, we subtracted the cumulative existing lake area that merged prior to calculating lake growth and net change. Individual lake net-change metrics are used to compute cumulative region-wide lake-area growth and loss metrics.

To map glacier-bed overdeepenings in the region, we combined the Copernicus GLO-30 Digital Elevation Model (50) (DEM) and previously modeled ice thickness estimates derived from observed ice velocities and the shallow-ice approximation (21). Imagery for GLO-30 was acquired between 2010 and 2015, while ice velocities used in Millan et al. (21) are from 2017 to 2018. The GLO-30 DEM was mosaicked, regridded to 50 m, and reprojected to NAD83/Alaska Albers (EPSG: 3338). The modeled ice thicknesses were mosaicked and reprojected and then subtracted from the surface DEM to generate a bed DEM. We created a filled bed surface using the “fillsinks” function in TopoToolbox (51) in Matlab version 2024b. In QGIS, we subtracted the filled bed DEM from the original bed DEM to map glacier-bed overdeepening spatial extents and magnitudes (48). To account for uncertainty in ice thickness estimates, we repeated the above workflow with revised ice thickness rasters that accounted for stated uncertainties. *SI Appendix*, Fig. S3 shows examples of overdeepenings using the best estimate of ice thickness, as well as ± ice thickness uncertainty.

To combine the lake growth and overdeepened rasters, we created a lake growth shapefile for two intervals: 2009–2018 and 2018–2024. The 2009 and 2018 lake outlines for the first interval are from Rick et al. (15) and the 2018 and 2024 lake outlines for the second interval were generated by this study. We used zonal statistics in QGIS to sample the overdeepened raster and calculate the area of glacier-bed overdeepening area within the lake growth shapefiles for each interval (48).

To assess future growth, we converted the overdeepened raster to a vector product using the Contour tool in ArcGIS Pro (Esri, Redlands, CA), thus producing an outline of glacier-bed overdeepenings (potential lake basins), and then used the Pairwise Erase tool to omit areas where 2018–2024 lake growth overlapped. We then completed a Spatial Join in QGIS to associate overdeepened polygons with lakes. When computing total overdeepened area, we removed duplicate overdeepenings when two or more lakes were joined to the same overdeepening (e.g., Bering Glacier).

For glacier surface velocity analysis, we utilize the Python API for accessing ITS_LIVE surface velocities derived from correlation of Landsat and Sentinel-2 optical satellite imagery (22). We extract point velocities near the glacier centerline several hundred meters upstream from the 2024 terminus position. In cases where glaciers had very wide termini or multiple terminal lobes, we extracted velocities at several locations. We removed velocity observations with uncertainty values > 40 m y^−1^ to improve time series data quality and limit results to image pairs separated by ≤120 to 300 d to mitigate temporal decorrelation. We manually reviewed velocity time series and selected a subset to illustrate the different styles of glacier velocity evolution over time. Glacier terminus positions were mapped for three glaciers illustrating the different modes of velocity response to lake growth (Fig. 4) using the Google Earth Engine Digitisation Tool (GEEDit) tool (52).

We analyzed published glacier elevation change rates (42) between 2010 and 2019. Using the Wilcoxson rank sum test (i.e., Mann–Whitney *U* test), we tested the null hypothesis that the data in the two samples (e.g., all glaciers and glaciers with ice-marginal lakes in each glacier area class) have equal medians, against the alternative that the medians are not equal. We determined that the medians were different if the null hypothesis was rejected at the 5% significance level.

## Supplementary Material

Appendix 01 (PDF)

## Data Availability

Data generated by this study, including the 2018 and 2024 ice-marginal lake shapefiles, the glacier-bed overdeepening rasters (accounting for ice thickness uncertainty), as well as per glacier and per lake glacier-bed overdeepening metrics, are available at https://doi.org/10.5061/dryad.rjdfn2zrc (48).
