# Supplementary material for: Rapid ice-marginal lake growth in Alaska driven by glacier retreat through bed overdeepenings
Source: Proc Natl Acad Sci U S A. 2026 Mar 9;123(12):e2513289123. doi: 10.1073/pnas.2513289123 (PMC13012058; doi:10.1073/pnas.2513289123)
Supplement: Supplementary file 1 — Appendix 01 (PDF) [file pnas.2513289123.sapp.pdf]

PNAS

Supporting Information for
Rapid ice-marginal lake growth in Alaska driven by glacier retreat through bed overdeepenings

Daniel McGrath[1*], Louis Sass[2], William H. Armstrong[3], Caitlyn Florentine[4], and Scott W. McCoy[5]

[1] Department of Geosciences, Colorado State University, Fort Collins, CO, USA, 80523
[2] U.S. Geological Survey Alaska Science Center, Anchorage, AK, USA, 99508
[3] Department of Geological and Environmental Sciences, Appalachian State University, Boone, NC, USA, 28608
[4] U.S. Geological Survey Northern Rocky Mountain Science Center, Bozeman, MT, USA, 59717
[5] Department of Geological Sciences and Engineering, University of Nevada, Reno, NV, USA, 89557
*Corresponding author: Daniel McGrath
Email: daniel.mcgrath@colostate.edu

Any use of trade, firm, or product names is for descriptive purposes only and does not imply endorsement by the U.S. Government.

This PDF file includes:

Figures S1 to S7
SI References

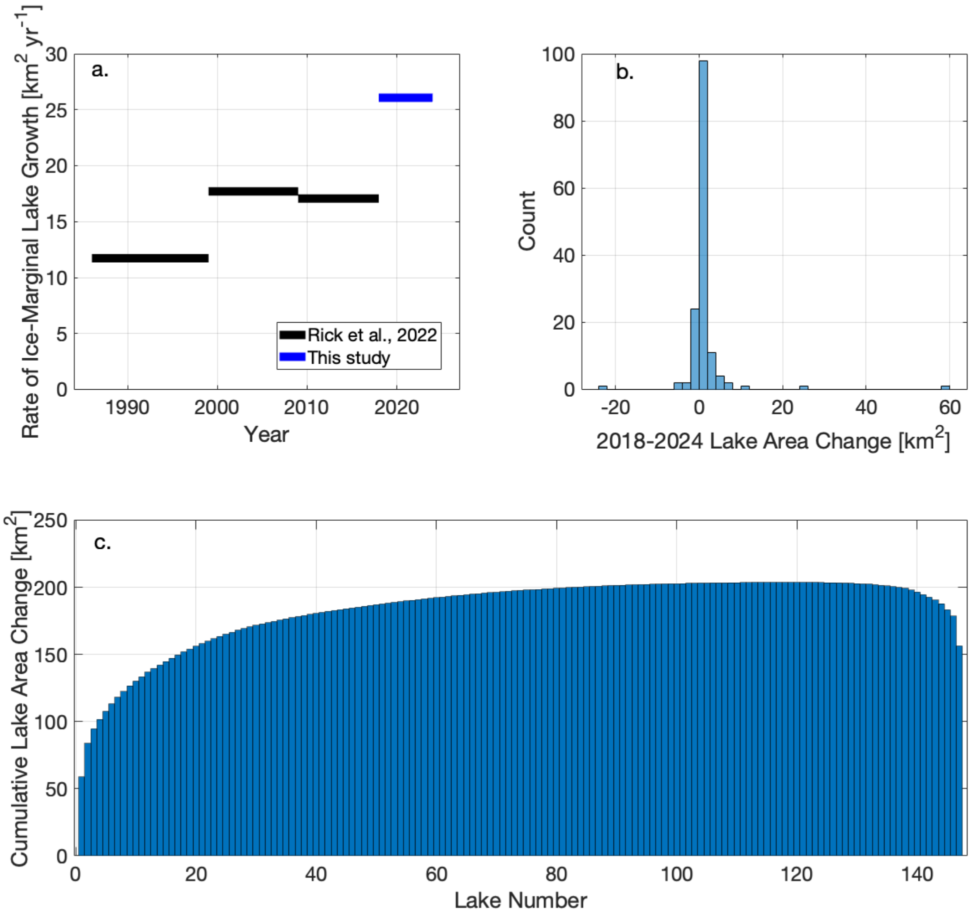

Fig. S1. Summary of ice-marginal lake area change in Alaska. a) Rate of ice-marginal lake growth from Rick et al. (1) and this study, b) Histogram of ice-marginal lake area change [km^2] between 2018 and 2024, c) Cumulative contribution of individual lakes to total lake area change [km^2] between 2018 and 2024.

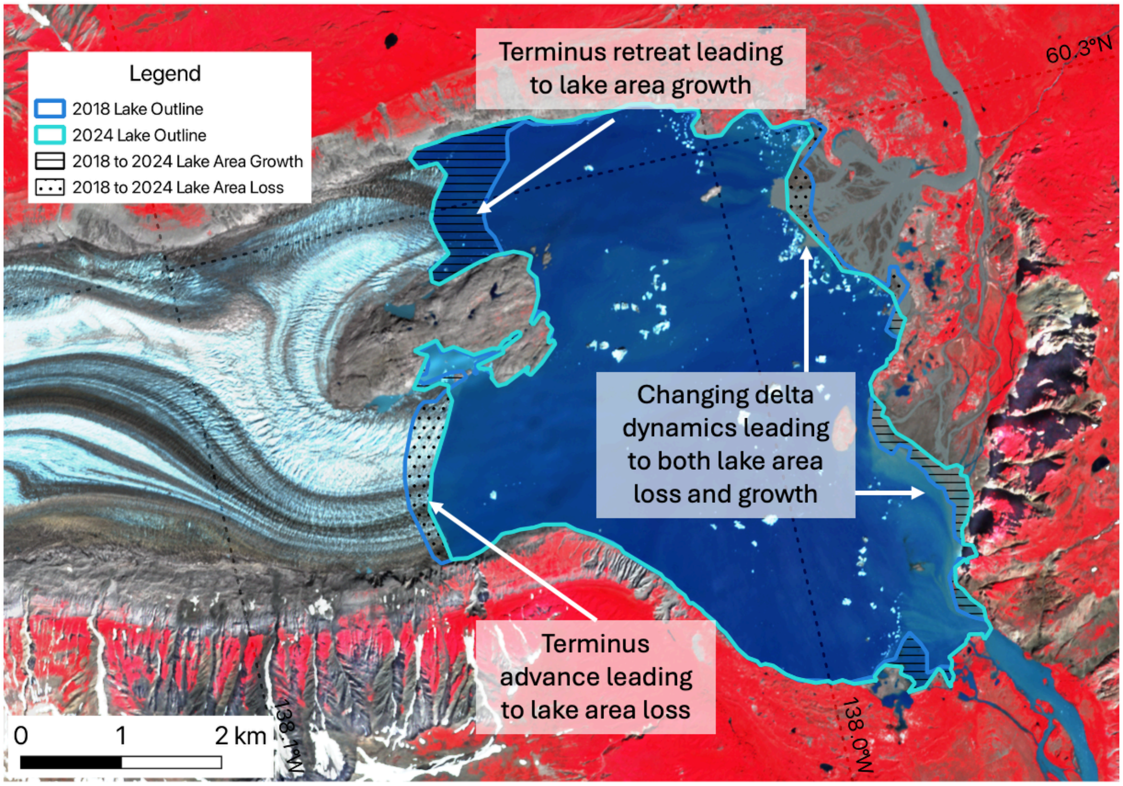

Fig. S2. Sentinel-2 false-color (bands 8,4,3) image from 2024 showing spatial patterns of lake area change proximal to Lowell Glacier. Variable terminus retreat and advance modify lake area, as does changing delta dynamics. For this example, the lake area net change was +0.87 km^2 with +1.56 km^2 of lake area growth.

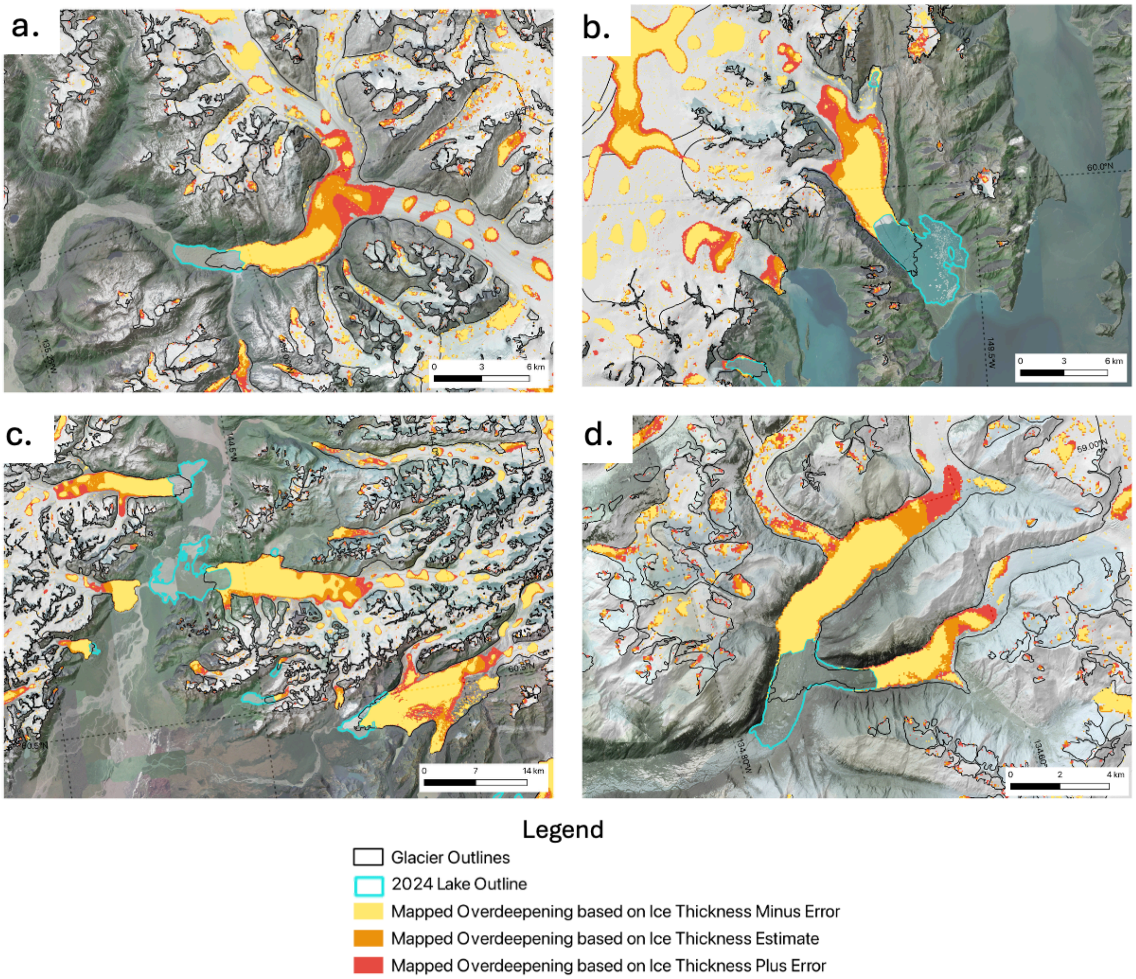

Fig. S3. Mapped glacier-bed overdeepening variations resulting in ice thickness error estimates for a) Meade, b) Bear, C) Allen, Miles and Martin River, and d) Field Glaciers. Imagery source: Esri, Vantor, Earthstar Geographics, and the GIS User Community. Hillshade source: USGS National Map 3D Elevation Program (3DEP).

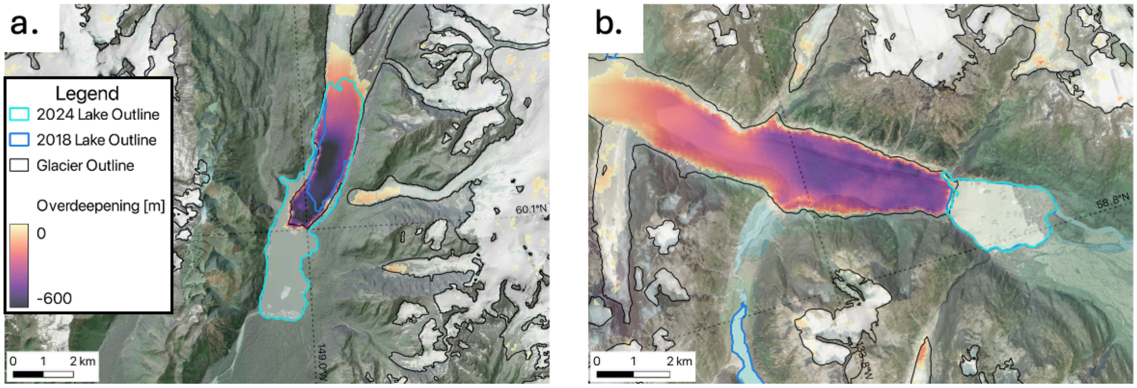

Fig. S4. Examples of contrasting future lake area growth potential for two lake-glacier systems. a) The ice-marginal lake adjacent to Ellsworth Glacier expanded by 4.3 km^2 between 2018 and 2024, but future expansion is limited as only 1.4 km^2 of glacier-bed overdeepening remains. b) The ice-marginal lake adjacent to Tulsequah Glacier remained stable between 2018 and 2024 but is connected to a >16 km^2 area of glacier-bed overdeepening, indicating a high potential for future growth. Imagery source: Esri, Vantor, Earthstar Geographics, and the GIS User Community. Hillshade source: USGS National Map 3D Elevation Program (3DEP).

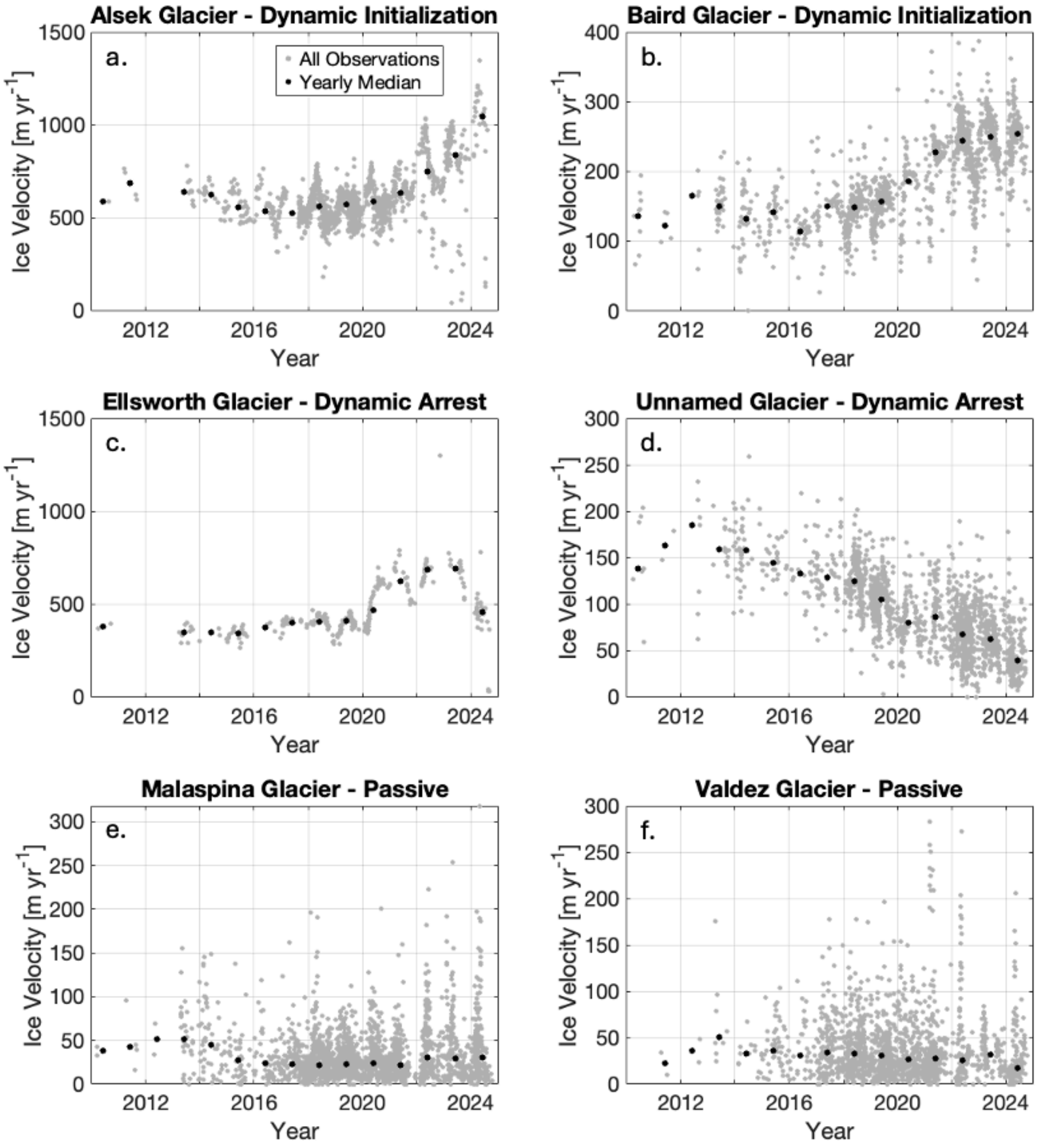

Fig. S5. Ice velocity time series for six glaciers illustrating the two phases of the dynamic mode a-b) initialization, c-d) arrest and, e-f) the passive mode. Grey points are all observations, black points are yearly median velocities. Unnamed glacier refers to RGI 6.0 – 01.05638.

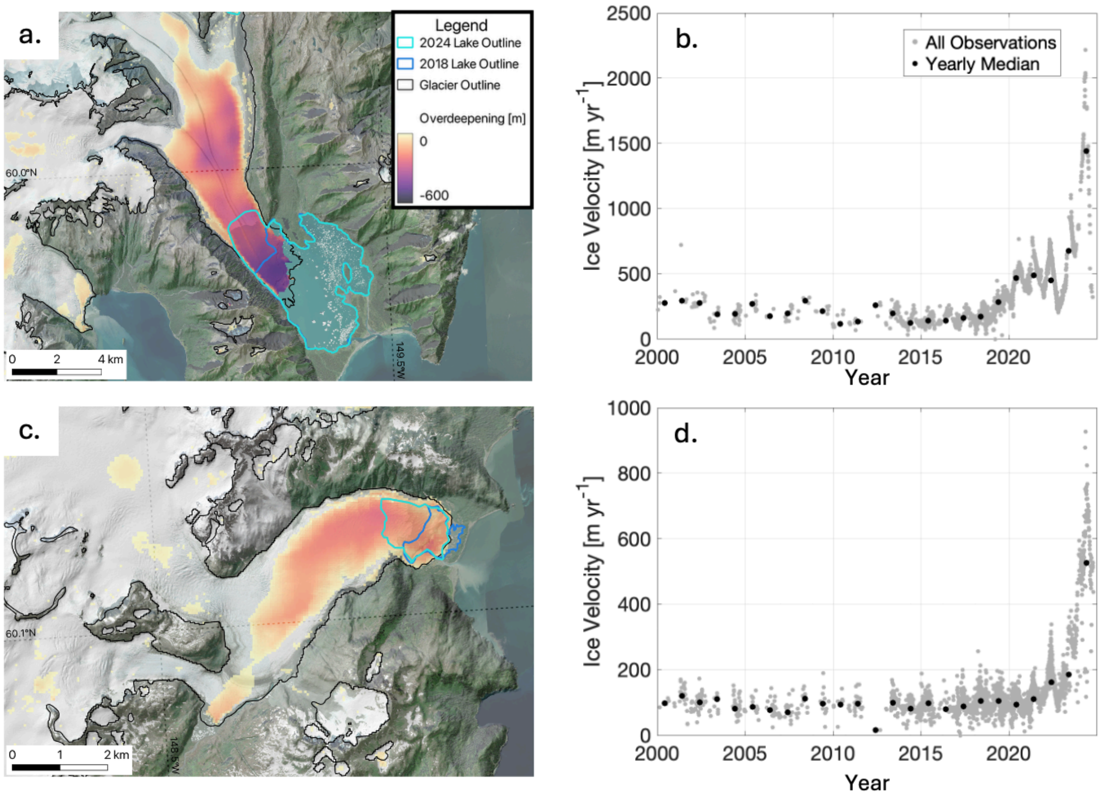

Fig. S6. Examples of two glaciers that experienced dynamic lake growth between 2018 and 2024 coinciding with ocean-water intrusion into the lake. a) Bear Glacier has expanded by 3.4 km^2 between 2018 and 2024, which b) coincided with a period of rapid acceleration. c) Bainbridge Glacier grew by 0.7 km^2 between 2018 and 2024, which d) coincided with a velocity increase of ~600 m yr^{-1}. Imagery source: Esri, Vantor, Earthstar Geographics, and the GIS User Community. Hillshade source: USGS National Map 3D Elevation Program (3DEP).

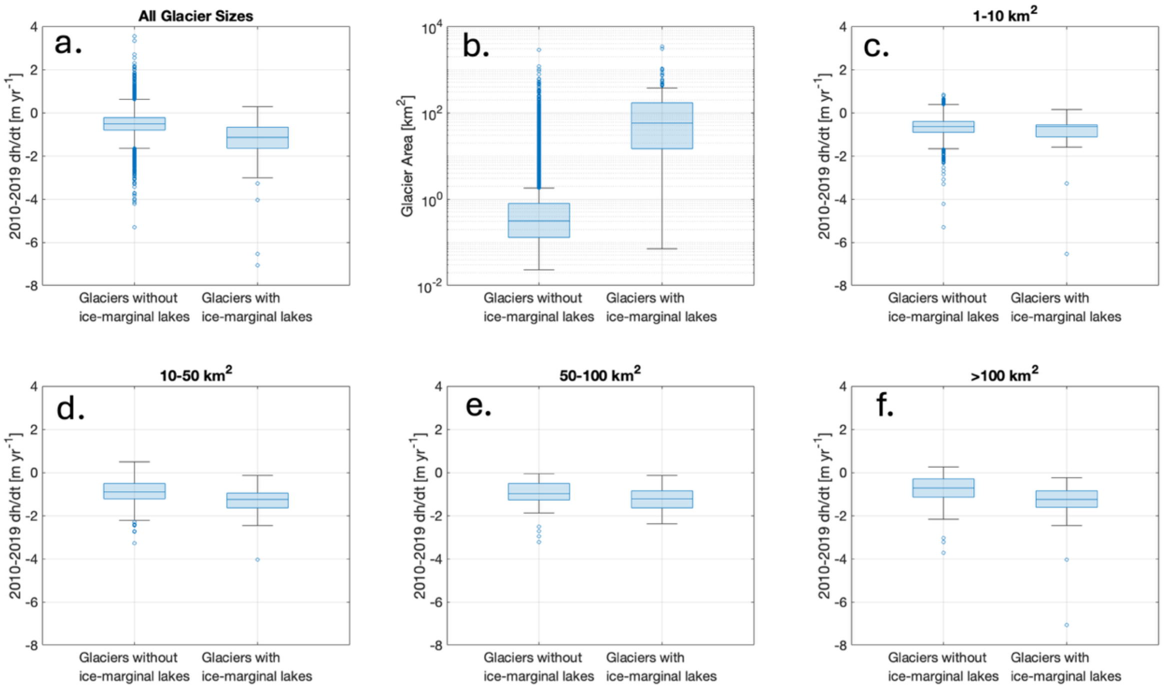

Fig. S7. Comparison of glacier elevation change between all glaciers and glaciers with ice-marginal lakes. a) Boxplot of glacier thinning rates from 2010–2019 for all glaciers in the region and those with ice-marginal lakes >0.5 km^2. b) Boxplots of glacier area for all glaciers in the region and those with ice-marginal lakes >0.5 km^2. Boxplots of glacier thinning rates from 2010–2019 for all glaciers and those with ice-marginal lakes >0.5 km^2 for c) 1–10 km^2, d) 10–50 km^2, e) 50–100 km^2 and f) >100 km^2. Differences were statistically significant for 10–50, 50–100, and >100 km^2 size classes, where glaciers with ice-marginal lakes thinned more than glaciers without ice-marginal lakes by −0.36 (34% difference), −0.26 (23% difference) and −0.53 m yr^{-1} (54% difference), respectively.

SI References

1. B. Rick, D. McGrath, W. Armstrong, S. W. Mccoy, Dam type and lake location characterize ice-marginal lake area change in Alaska and NW Canada between 1984 and 2019. *Cryosphere* 16, 297–314 (2022).
